# Supplementary figures and images for: Advice to Clinicians on Communication from Adolescents and Young Adults with Cancer and Parents of Children with Cancer
Source: Children (Basel). 2022 Dec 21;10(1):7. doi: 10.3390/children10010007 (PMC9856802; doi:10.3390/children10010007)

**Figure S1. Parent characteristics**

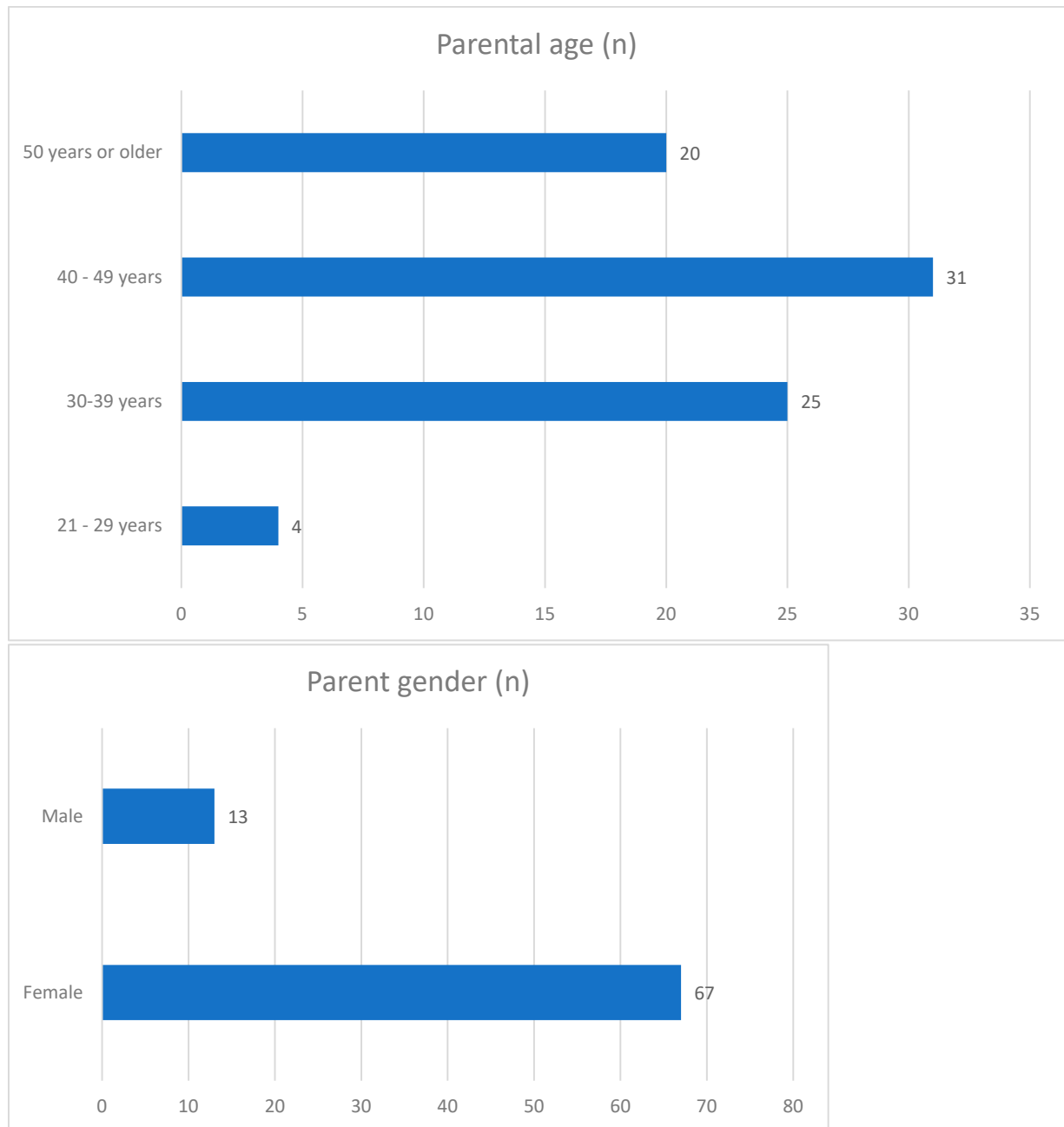

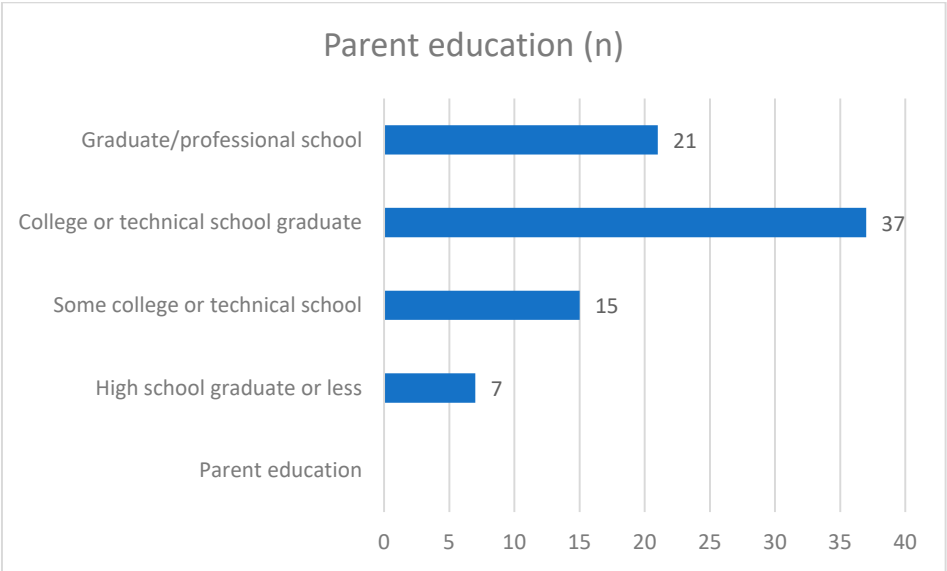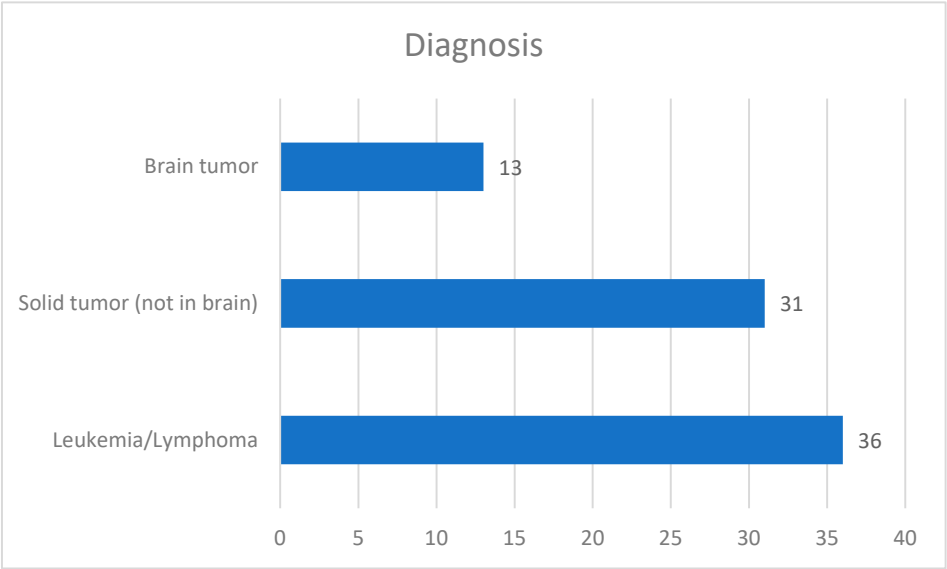

**Figure S2. AYA characteristics**

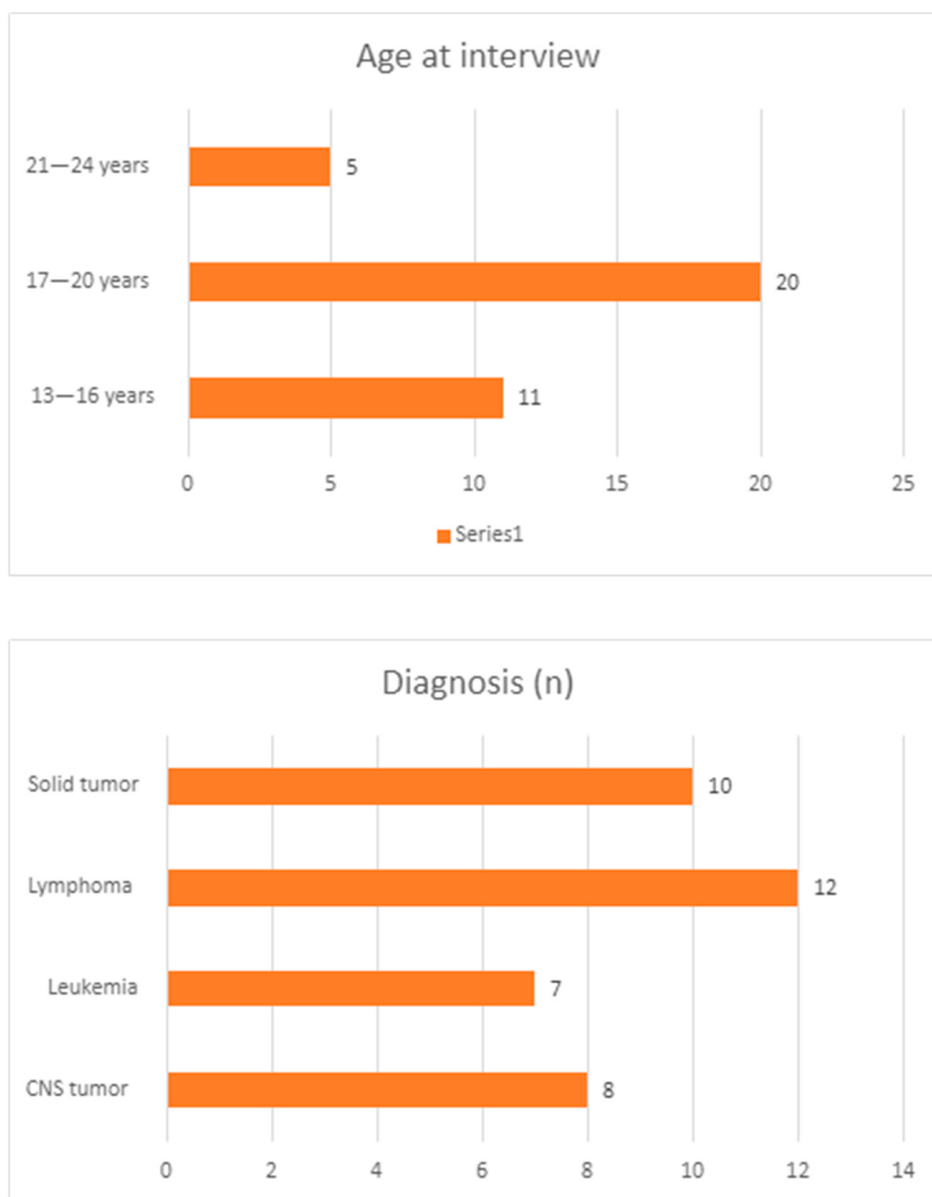

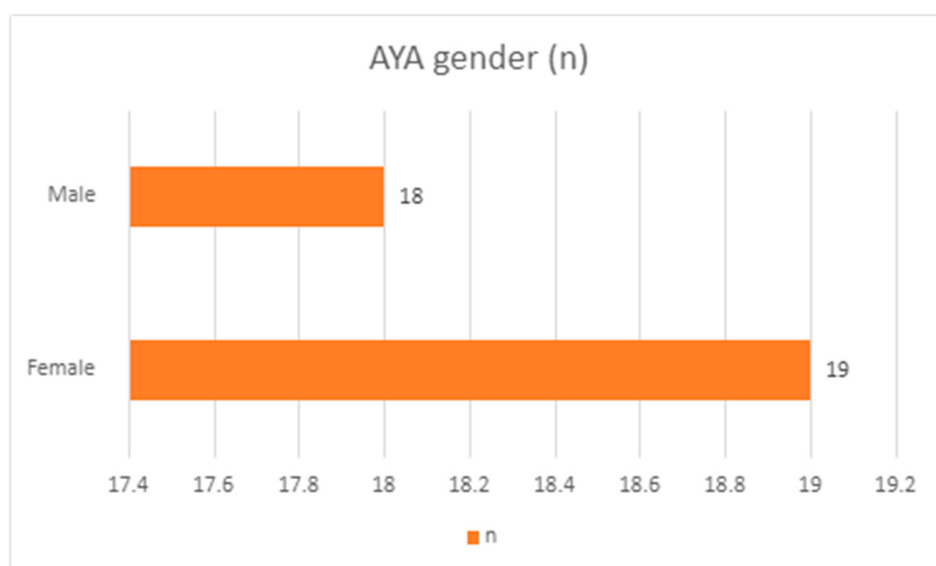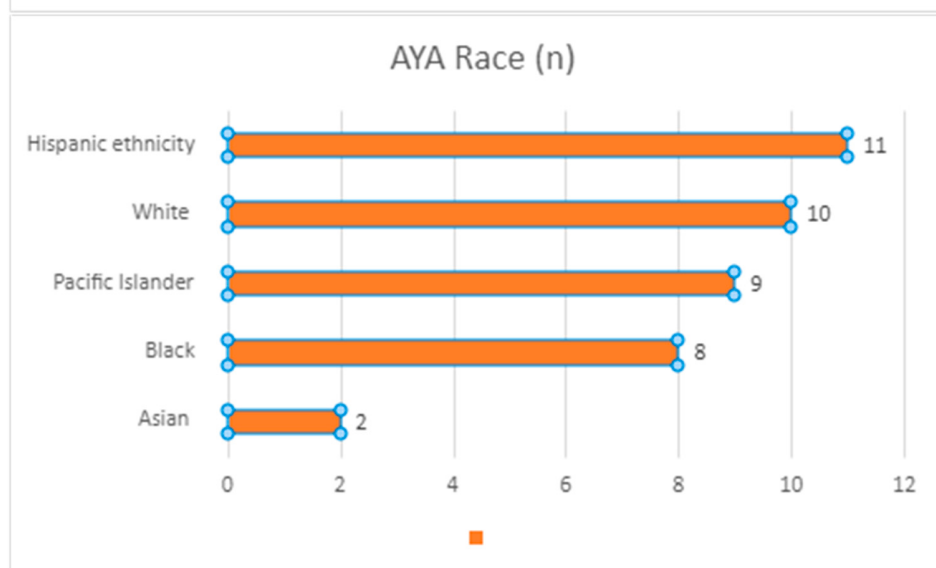

Supplement: Supplementary file 1 [file children-10-00007-s001.zip › children-2075509-supplementary.pdf]
